# Case report: Completely unroofed coronary sinus with a left superior vena cava draining into the left atrium studied by cardiovascular magnetic resonance

**DOI:** 10.4103/0971-3026.69359

**Published:** 2010-08

**Authors:** Vimal Raj, Sanjiv Joshi, Yuen Chi Ho, Philip J Kilner

**Affiliations:** Cardiovascular Magnetic Resonance Unit, Royal Brompton Hospital and Imperial College, Sydney Street, SW3 6NP, London, United Kingdom

**Keywords:** Atrial septal defect, cardiovascular magnetic resonance imaging, pulmonary hypertension, unroofed coronary sinus

## Abstract

A persistent left superior vena cava (LSVC) draining through a dilated coronary sinus into the right atrium is a relatively common congenital cardiovascular anomaly. It is readily identified by cardiovascular magnetic resonance (CMR). However, a LSVC draining into the left atrium (LA) and associated with unroofing of the coronary sinus, with resulting interatrial communication, is rare and may have important clinical consequences. As with any large atrial septal defect, it can be associated with a higher than expected incidence of pulmonary arterial hypertension, systemic embolization, and brain abscesses. In this report, we present a case of a completely unroofed coronary sinus with a persistent LSVC draining directly into the LA and illustrate the role of CMR in the diagnosis and evaluation of such anomalies.

## Introduction

A persistent left superior vena cava (LSVC) draining through a dilated coronary sinus into the right atrium is a relatively common congenital cardiovascular anomaly. It is readily identified by cardiovascular magnetic resonance (CMR). However, a LSVC draining into the left atrium (LA) and associated with unroofing of the coronary sinus, with resulting interatrial communication, is rare and may have important clinical consequences. As with any large atrial septal defect, it can be associated with a higher than expected incidence of pulmonary arterial hypertension, systemic embolization, and brain abscesses. In this report, we present a case of a completely unroofed coronary sinus with a persistent LSVC draining directly into the LA and illustrate the role of CMR in the diagnosis and evaluation of such anomalies.

## Case Report

A 76-year-old gentleman with a history of obstructive airways disease and obstructive sleep apnea was found to be hypoxic during a routine colonoscopy procedure. To evaluate his lungs and hypoxia further, he was referred for a CT pulmonary angiogram (CTPA). This was performed on a third generation multislice scanner (GE LightSpeed RT, GE Medical Systems, Milwaukee, WI) which demonstrated anomalous drainage of a left superior vena cava (LSVC) into the left atrium (LA) with features of pulmonary arterial hypertension and a possible atrial septal defect (ASD). Transthoracic echocardiography (TTE) confirmed pulmonary hypertension, but was unable to demonstrate either an ASD or the anomalous drainage due to suboptimal views in this rather large patient. Cardiac catheter study with oxygen saturations performed on the same day demonstrated a shunt fraction of 1. Subsequent cardiovascular magnetic resonance (CMR) imaging was requested to further delineate the anatomy and physiology. This was performed on a 1.5-T system (Avanto, Siemens, Germany).

**Figure 1 (A-E):**
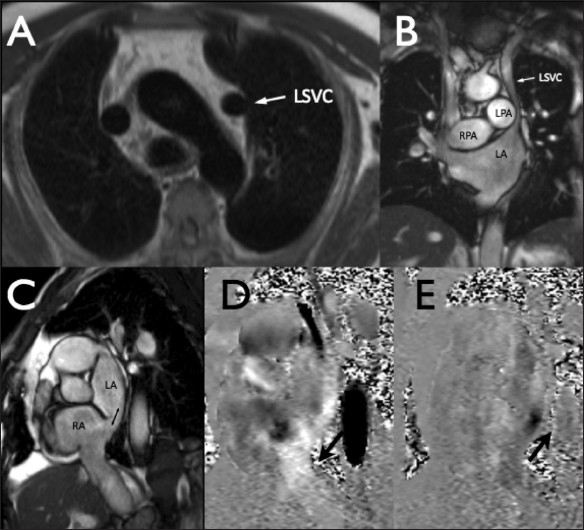
Transaxial (A) and coronal (B) images show a left superior vena cava (LSVC) draining into the left atrium (LA). Dilated right and left pulmonary arteries (RPA and LPA) can also be seen. Atrial short-axis (C) and the velocity-encoded images (D - systole, E - diastole) show the site of bidirectional flow between the right and the left atria (double-headed arrow in C)

Transaxial multislice scout images [Figure 1A] and multiple steady-state free precession cine images in left ventricular long- and short-axis planes were acquired. A coronal cine was acquired to show the LSVC relative to the LA [Figure 1B]. In view of the suspected ASD, a contiguous stack of atrial short-axis cine images was also acquired, parallel to the ventricular short axis [Figure 1C], and breath-hold phase-contrast velocity mapping was performed through planes transecting the aorta with a velocity encoding (VENC) of 150 as well as transecting the main pulmonary artery (VENC 150) and in a plane aligned with flow through the suspected interatrial communication [Figure 1D and E].

**Figure 2 (A-C):**
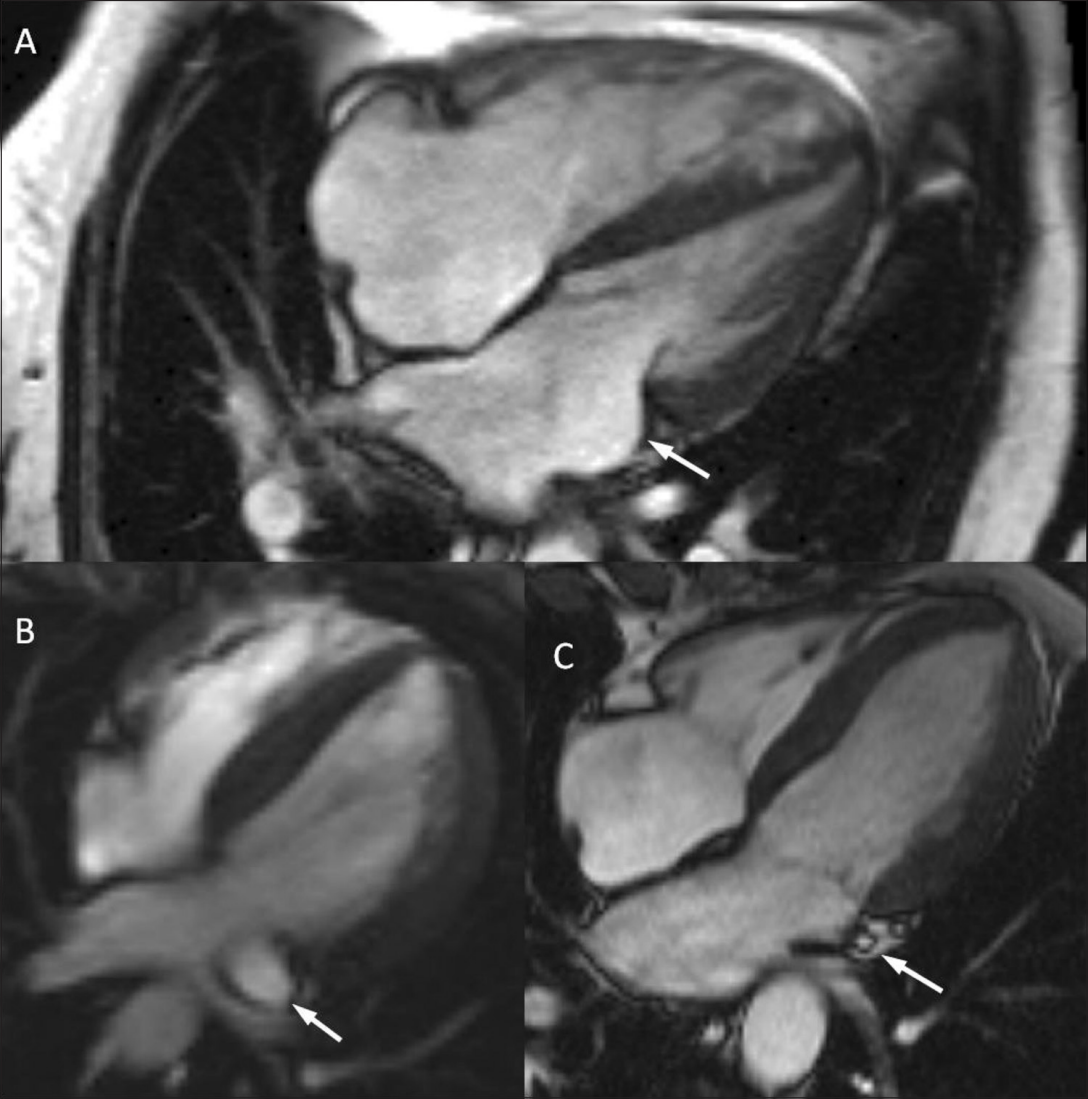
(A) Four-chamber image of the patient shows absence of a dilated coronary coronary sinus (arrow) which, given the presence of the LSVC, would be expected to pass through this plane in the region of the arrow. The image also shows relative dilatation of the right atrium and right ventricle. (B) For comparison, the four-chamber image of another patient with a persistant LSVC shows a dilated coronary sinus (arrow) which drains into the right atrium. (C) For further comparison, four-chamber image of a healthy volunteer shows a normal-sized coronary sinus (arrow)

The images and velocity maps showed evidence of a completely unroofed coronary sinus. Given the fact that there was a left as well as a right SVC, visibility of its continuation into a dilated coronary sinus would have been expected in the three- or four-chamber long-axis cine images, but this was not the case [Figure 2]. Bidirectional flow through the associated interatrial communication, which was about 15 mm in diameter, was visible in the atrial short-axis cine images and the corresponding velocity acquisition [Figure 1D and E]. The through-plane velocity mapping measured 4.4 l/min in the pulmonary trunk and 6.9 l/min in the ascending aorta, indicative of a net right-to-left shunt with Qp : Qs = 0.6 : 1. The right ventricle was dilated and moderately hypertrophied, with a low ejection fraction of 25%, and there was systolic flattening of the interventricular septum. Biventricular volume measurements showed a larger left ventricular stroke volume than right ventricular stroke volume, which was in accordance with the presence of a right-to-left shunt. The pulmonary arteries were dilated (main PA = 41 mm) and showed limited systolic expansion on cine images. The combined findings were consistent with pulmonary arterial hypertension.

## Discussion

This case is salutary. Earlier detection of the interatrial communication and effective surgical closure with reconnection of the LSVC to the right atrium might have avoided the development of pulmonary vascular disease.

Unroofed coronary sinus defects comprise less than 1% of all ASDs.[1,2] The unroofing of the coronary sinus can either be partial or complete. Unroofed coronary sinus is associated with other congenital heart diseases, of which persistent LSVC is one.[3] Persistent LSVC can be seen in up to 0.5% of the general population. When present, the majority drain into the coronary sinus; about 8% drain directly into the LA.[4]

Unroofed coronary sinus has been classified morphologically into four types by Kirklin and Barratt-Boyes,[3,5] which are as follows:

Type I: Completely unroofed, with LSVC
Type II: Completely unroofed, without LSVC
Type III: Partially unroofed midportion
Type IV: Partially unroofed terminal portion

The above case is typical of type I. CMR demonstrated the anomolous anatomy with the bidirectional flow across the defect and quantified the net right-to-left shunt. It confirmed the features of pulmonary hypertension and quantified the severity of right heart failure.

The recognition of this anomaly is important as it can predispose to a number of complications, including systemic emboli, brain abscess, premature death, right heart failure, left ventricular dysfunction, tricuspid and mitral regurgitation, atrial flutter or fibrillation, and pulmonary and systemic hypertension.[1]

The diagnosis of unroofed coronary sinus has previously been made by cardiac angiography, surgery, or even necropsy.[6] Although echocardiography has developed as one of the main assessment tools, the diagnosis of unroofed coronary sinus remains tricky and unsatisfactory, especially in patients with large body habitus who might have poor echo windows for TTE. Transoesophageal echocardiogram (TOE) largely provides better visualization of cardiac structures and is superior in demonsrating shunts. However, TOE is an invasive procedure that requires some premedication and/or sedation in a large number of patients. One surgical series showed that the condition was correctly diagnosed preoperatively in only 6 out of 11 cases.[3]

The usefulness of CMR in the assessment and diagnosis of this entity in adult patients is relatively underpublished.[7] Its value is likely to depend on the radiologist’s awareness of the condition and on whether appropriately thorough acquisition protocols have been followed. In the presence of a LSVC, a region of unroofing is not easy to exclude, although appropriately acquired and analyzed aortic and pulmonary flow measurements provide a valuable measurement of shunt flow. Compared with cardiac angiography, CMR is noninvasive, does not involve exposure to ionizing radiation, and avoids the need for contrast injection (unlike CT scan). CMR allows relatively comprehensive functional and anatomical assessments in a single investigation.

In summary, this case illustrates the usefulness of CMR in demonstrating the abnormal anatomy and the pathophysiology associated with the rare congenital anomaly of unroofed coronary sinus.
